# Towards big data science in the decade ahead from ten years of InCoB and the 1st ISCB-Asia Joint Conference

**DOI:** 10.1186/1471-2105-12-S13-S1

**Published:** 2011-11-30

**Authors:** Shoba Ranganathan, Christian Schönbach, Janet Kelso, Burkhard Rost, Sheila Nathan, Tin Wee Tan

**Affiliations:** 1 Department of Chemistry and Biomolecular Sciences and ARC Centre of Excellence in Bioinformatics, Macquarie University, Sydney NSW 2109, Australia; 2 Department of Biochemistry, Yong Loo Lin School of Medicine, National University of Singapore, Singapore 117597; 3 Department of Bioscience and Bioinformatics, Graduate School of Computer Science and Systems Engineering, Kyushu Institute of Technology, Fukuoka 820-8502, Japan; 4 Department of Evolutionary Genetics, Max Planck Institute for Evolutionary Anthropology, 04103 Leipzig, Germany; 5 Department for Bioinformatics and Computational Biology, Faculty for Informatics, Technical University Munich, 85748 Garching, Germany; 6 School of Biosciences and Biotechnology, Faculty of Science and Technology, Universiti Kebangsaan Malaysia, 43600 UKM Bangi Selangor D.E., Malaysia; 7 Malaysia Genome Institute, Jalan Bangi, 43000 Kajang Selangor D.E., Malaysia

## Abstract

The 2011 International Conference on Bioinformatics (InCoB) conference, which is the annual scientific conference of the Asia-Pacific Bioinformatics Network (APBioNet), is hosted by Kuala Lumpur, Malaysia, is co-organized with the first ISCB-Asia conference of the International Society for Computational Biology (ISCB). InCoB and the sequencing of the human genome are both celebrating their tenth anniversaries and InCoB’s goalposts for the next decade, implementing standards in bioinformatics and globally distributed computational networks, will be discussed and adopted at this conference. Of the 49 manuscripts (selected from 104 submissions) accepted to *BMC Genomics* and *BMC Bioinformatics* conference supplements, 24 are featured in this issue, covering software tools, genome/proteome analysis, systems biology (networks, pathways, bioimaging) and drug discovery and design.

## Introduction

InCoB (International Conference on Bioinformatics), the official conference of the Asia-Pacific Bioinformatics Network (APBioNet) [1] is celebrating its 10th anniversary this year, as a joint conference with the first ISCB-Asia meeting of the International Society for Computational Biology (ISCB) [2], at Kuala Lumpur, Malaysia. Since the first 2002 meeting in Bangkok, Thailand, InCoB serves as one of the largest bioinformatics conferences in the Asia-Pacific region, publishing submissions as research papers in conference supplements of international PubMed-indexed open-access impact factor journals, since 2006.

As InCoB’s 10th anniversary coincides with that of the human genome, the results of the systematic genome-wide sequencing applied to medical purposes, are appearing in the literature according to Collins [3], although Venter [4] believes we still have a long way to go before genome sequencing reaches its full potential. While genomic data is reaching tsunami proportions [5], its clinical applications are seen as a “slowly rising tide” [6]. Perhaps, as Trelles et al. [7] suggest, we are not yet ready for Big Data science. As succinctly summarized by Pennisi [8], while sequencing technologies have become more and more affordable, the challenges of storing, comparing and analyzing the data appear to persist, despite computational solutions proposed by Schadt et al. [5] and Zhou et al. [9].

We see these issues as challenges for the next decade, with cloud [10] and grid computing (reviewed in the first InCoB2006 publication [11]), gearing up for the data deluge, and data interchange standards becoming better established and adopted. In the Asia-Pacific, large scientific consortia are addressing personal genomic questions of local interest, such as the Pan Asian SNP initiative [12], which has provided a possible route for human migration into Asia [13]. We have to figure out how to build the resources for hosting Big Data in our own regions, with well organized and structured access to this Big Data, as a first step. Concurrently, with pre-existing computational resources are already available to our researchers, we need to motivate our researchers to ask the right questions of this Big Data and generate meaningful results.

For InCoB/ISCB-Asia 2011, we have therefore introduced dedicated sessions in Standards in Bioinformatics, following a keynote address on Biocuration by Gaudet and BioCloud/Grid Computing for Sharing Bioinformatics Resources. From APBioNet, we will present the Minimum Information About a Bioinformatics Investigation initiative (MIABi) [14] as well as a status update on BioDB100, the 100 MIABI-compliant BioDatabases initiative. We will also launch our BioSW100, the 100 MIABi-compliant BioSoftware initiative and invite the community to contribute to these ongoing projects, for provide Big Data in standardized format for developing distributed workflows that are grid- and cloud-enabled, to bring “bioinformatics to the bedside” a step closer to reality. We also noticed that since InCoB2008, accepted papers have focused on identifying target disease genes using networks, pathways and systems biology approaches as well as drug design and discovery, enabling translational bioinformatics.

## Submissions and review for InCoB/ISCB-Asia 2011

Of the 104 submissions received this year, we accepted 24 articles for *BMC Bioinformatics* (this issue), 25 for *BMC Genomics *[15] and four for *Immunome Research *[16], an independent bioinformatics-driven immunology journal. Details of the reviewing process are presented in the *BMC Genomics* introduction article [15], with at least three reviews for each submission (see Additional File 1 of ref. [15] for a list of reviewers) and in the majority of the acceptable papers going through two rounds of reviews. The submitted articles originated from 19 countries with East Asia, South-East Asia and South Asia accounting for 83% of the submissions and 82% of the acceptances (details in Additional File 2 of ref. [15]), reinforcing the strong regional support for InCoB and ICSB-Asia from the region.

The challenges of developing bioinformatics research tools and applying them to the areas of genome and proteome analysis, systems biology (networks, pathways and bioimaging) and structure-based drug design and discovery are presented in this issue.

## Software tools

Firdaus-Raih et al. [17] have a novel graph theoretical method to identify highly stable base triplets in RNA structures, while Benso et al. [18] have proposed simple decision rules in R, to classify gene expression data. PTIGS-IdIt [19] provides a novel approach for plant species identification using DNA barcoding technology, with HabiSign [20] for habitat-specific metagenome analysis. A webserver for predicting dinucleotide-specific RNA-binding sites is presented by Fernandez et al. [21], while PB1-F2 Finder by DeLuca et al. [22] can scan influenza viral sequences for specific RNA encoding regions. Protein analysis methods include support vector machine (SVM) models to predict RNA-binding residues (Choi and Han [23]) and to differentiate between carboxylation and non-carboxylation sites (Lee et al. [24]) while Nair et al. [25] have combined several machine learning approaches to predict amyloidogenic regions.

## Genome and proteome analysis

Kim et al. [26] have evaluated the performance of several matrix factorization methods for clustering gene expression data, while Mallek et al. [27] have compared the efficacy of four predictive models for estimating chlorophyll-a concentrations in environmental samples. Choi et al. [28] have used molecular dynamics to predict the functionality of a hypothetical pathogen protein.

## Systems biology: pathways, networks and imaging

Networks of biomolecules and pathways provide a deep understanding of the mode of action of biological systems. Poirel et al. [29] report a network approach to function enrichment. Soh et al. [30] have identified disease subnetworks from gene expression data. While Hsu et al. [31] have explored consistency in gene interaction networks, Rajapakse and Mundra [32] have proposed models for estimating the stability of gene interaction networks. Lee et al. [33] present an application of protein interaction networks to neurological disorders and Liu et al. [34] have proposed a possible initiation of the Wnt signaling pathway using a conformational simulation approach.

Bioimaging captures biological processes in real-time. Du et al. [35] have demonstrated that automated cell cycle phase classification can be applied to monitor *in vivo* cellular processes, while Veronika et al. [36] have correlated membrane dynamics with cell motility.

## Structure-based drug design and discovery

With the availability of 3D structures for drug targets, Grover et al. [37] have proposed a possible mechanism for the action of the herbal drug, withaferin A, used in the treatment of herpes simplex virus, Tambunan et al. [38] explored modifications improve the efficacy of a known histone deacetylase inhibitor of the oncogenic human papilloma virus, while Lim et al. [39] have used virtual screening to identify candidate drug molecules for dengue virus methyl transferase. Khanna and Ranganathan [40] have proposed a novel set of antiparasitic compounds using an SVM approach.

## Conclusion

We are encouraged by the robust support for InCoB and ISCB-Asia, arising from the strong representation from the region in the accepted papers and posters. We believe the region is well poised to exploit the latest technological advances in high-throughput sequencing, data dissemination as well as computational analyses, to usher in an era of personalized medicine. To ensure that these activities are compliant with international standards, we have included biocuration and standards as a new initiative in InCoB/ISCB-Asia 2011 and will provide updates on APBioNet’s BioDB100 and BioSW100 projects at InCoB2012.

## Competing interests

The authors declare that they have no competing interests.

## Authors' contributions

SR and CS (Program Committee Co-chairs) wrote the introduction and managed the review and editorial processes. CS, SR, JK (Chair, ISCB Conferences Committee), BR, TWT and SN (Conference Chair) jointly contributed to the scientific program development and its implementation. TWT supported the post-acceptance manuscript processing.
